# MALDI-TOF MS identification of *Anopheles gambiae* Giles blood meal crushed on Whatman filter papers

**DOI:** 10.1371/journal.pone.0183238

**Published:** 2017-08-17

**Authors:** Sirama Niare, Lionel Almeras, Fatalmoudou Tandina, Amina Yssouf, Affane Bacar, Ali Toilibou, Ogobara Doumbo, Didier Raoult, Philippe Parola

**Affiliations:** 1 Aix Marseille Université, Unité de Recherche en Maladies Infectieuses et Tropicales Emergentes (URMITE), UM63, CNRS 7278, IRD 198 (Dakar, Sénégal), Inserm 1095, AP-HM, IHU Méditerranée Infection, Marseille, France; 2 Malaria Research and Training Center, DEAP/FMOS, UMI 3189, University of Science, Techniques and Technology, Bamako, Mali; 3 Unité de Parasitologie et d’Entomologie, Département des Maladies Infectieuses, Institut de Recherche Biomédicale des Armées, Marseille, France; 4 Malaria Control Program, Moroni, Union of the Comoros; National Institute for Communicable Diseases, SOUTH AFRICA

## Abstract

**Background:**

Identification of the source of mosquito blood meals is an important component for disease control and surveillance. Recently, matrix-assisted laser desorption/ionization time-of-flight mass spectrometry (MALDI-TOF MS) profiling has emerged as an effective tool for mosquito blood meal identification, using the abdomens of freshly engorged mosquitoes. In the field, mosquito abdomens are crushed on Whatman filter papers to determine the host feeding patterns by identifying the origin of their blood meals. The aim of this study was to test whether crushing engorged mosquito abdomens on Whatman filter papers was compatible with MALDI-TOF MS for mosquito blood meal identification. Both laboratory reared and field collected mosquitoes were tested.

**Material and methods:**

Sixty *Anopheles gambiae* Giles were experimentally engorged on the blood of six distinct vertebrate hosts (human, sheep, rabbit, dog, chicken and rat). The engorged mosquito abdomens were crushed on Whatman filter papers for MALDI-TOF MS analysis. 150 Whatman filter papers, with mosquitoes engorged on cow and goat blood, were preserved. A total of 77 engorged mosquito abdomens collected in the Comoros Islands and crushed on Whatman filter papers were tested with MALDI-TOF MS.

**Results:**

The MS profiles generated from mosquito engorged abdomens crushed on Whatman filter papers exhibited high reproducibility according to the original host blood. The blood meal host was correctly identified from mosquito abdomens crushed on Whatman filter papers by MALDI-TOF MS. The MS spectra obtained after storage were stable regardless of the room temperature and whether or not they were frozen. The MS profiles were reproducible for up to three months. For the Comoros samples, 70/77 quality MS spectra were obtained and matched with human blood spectra. This was confirmed by molecular tools.

**Conclusion:**

The results demonstrated that MALDI-TOF MS could identify mosquito blood meals from Whatman filter papers collected in the field during entomological surveys. The application of MALDI-TOF MS has proved to be rapid and successful, making it a new and efficient tool for mosquito-borne disease surveillance.

## Introduction

Malaria is a mosquito-borne infectious disease affecting humans and some animals, which is caused by parasitic protozoans belonging to the *Plasmodium* genus. Six species are known to infect humans [1,2]. Although malaria mortality rates have decreased in recent years, the disease remains one of the world’s three biggest killers, particularly in sub-Saharan Africa [3]. According to the World Health Organization (WHO), malaria is responsible for more than 400,000 deaths per year, with 90% of deaths occurring in Africa [1].

Female mosquitoes of the *Anopheles* genus, around 50 species have been described as vectors of malaria transmission. In sub-Saharan Africa, mosquitoes from the *Anopheles gambiae* complex are the main vectors of malaria transmission [4]. The malaria parasite is transmitted to human during the blood meals of infected female mosquitoes of the *Anopheles* genus. For a better understanding of the dynamics of malaria transmission, identification of the sources of blood meals is critical to assessing the risk of human exposure and determine the *Anopheles* trophic preference patterns. For example, evaluation of the *Anopheles* trophic preference patterns would make it possible to determine anthropophilic behavior in given malaria transmission areas [5]. An understanding of the origin of the mosquito host blood will enable the implementation of better control strategies to reduce the mosquito population and, therefore, transmission of disease. Measuring the proportion of blood meals taken from humans by mosquitoes (the human blood index) is an important component in vectorial capacity estimation.

Several techniques are used to identify the source of mosquito blood meals, including serological tools such as precipitin and ELISA tests [6,7]. These methods present several limitations, in terms of antibody specificity and the sensitivity of accurately identifying a large range of host bloods [8–10]. Gradually, molecular biology approaches were adopted as a means of identifying mosquito blood sources. DNA sequence amplification of host blood from mosquitoes has enabled greater sensitivity and specificity in relation to identification [11]. Nevertheless, the process can be costly and time consuming.

Recently, matrix-assisted laser desorption ionization–time of flight mass spectrometry (MALDI-TOF MS) has been used as alternative and rapid tool for mosquito blood meal identification [12]. The MALDI-TOF tool is routinely used to identify microorganisms both from cultures and directly from biological samples. MALDI-TOF MS presents many advantages, including the fact that it is such as reliable and cost-effective technique in comparison to conventional phenotypic and molecular methods for identifying microorganisms. Given the increase in emerging diseases such as arbovirus and parasitic diseases transmitted by mosquitoes, the development of new strategies could be essential to vector control. MALDITOF MS was recently used as an innovative, rapid, and reliable tool in entomological studies [13]. However, storage methods and the length of preservation can alter MALDI-TOF spectra for identification purposes [14]. MALDI-TOF MS has also emerged in medical entomology as an effective tool for identifying arthropods. The MS protein extracts from whole specimens or body parts of arthropods have been demonstrated to be an efficient and reproducible method of arthropod identification using MALDI-TOF MS [13]. In a previous study, the proof-of-concept of MALDI-TOF MS was successfully demonstrated through the identification of mosquito blood meals from recently fed mosquitoes. The abdomen proteins extracted from *Anopheles gambiae* which were either non-engorged or artificially engorged on seven distinct types of vertebrate blood, including human blood, have been suggested at by MALDI-TOF MS analysis. By comparing MALDI-TOF spectra from freshly engorged mosquito abdomens collected between 1 and 24 hours post-feeding, we were able to distinguish blood meal origins [12].

During entomological surveys, Whatman’s filter papers (WFPs) are frequently used in the field to collect and preserve the blood meals of arthropods. For this purpose, we proposed using the Whatman filter paper as a means of preserving samples and adapting to the field conditions. The abdomens of engorged female mosquitoes were crushed on WFPs, which were then transported to the laboratory for analysis and blood identification [15,16]. The advantage of WFPs is that the specimens can be individually crushed and stored at ambient temperature. The WFP collection method appears to be a simple and economical method for storage and preservation [17].

The aim of this study was to test the sensitivity and specificity of MALDI-TOF MS in identifying mosquito blood meal sources from the abdomens of blood-fed females crushed on WFPs. *Anopheles gambiae* Giles mosquitoes were experimentally fed with various host bloods, before MALDI-TOF analysis. We also attempted to see whether MALDI TOF could be used in the field in areas where malaria is endemic.

## Materials and methods

### Ethical statements and blood sampling

This study was conducted in accordance with the World Health Organization’s (WHO) Good Laboratory Practice guidelines and documents on blood sample handling procedures [18]. The mosquitoes were reared in line with the International Conference on Harmonization/Good Laboratory Practices (ICH/GLP) procedures. Laboratory technicians and students were trained and certified in animal- and insectaria-based experiments. The laboratory procedures adopted in this study were approved by the human and animal ethics committees of the Aix-Marseille University Institutional Animal Care and Use Committee. The animal blood (chicken, rabbit, sheep, rat, and dog) was provided by local animal houses and handled as per French Decree No. 8 87–848 (October 19, 1987, Paris). Human blood was obtained from the Etablissement Français du Sang (EFS), under existing agreements between the URMITE laboratory and the EFS [12]. In the field, all procedures adopted in this study were approved by the Comoros ethics committee and carried out with the agreement of the Ministry of Health and leaders of the villages selected for mosquito collection as part of the Programme National de Lutte contre le Paludisme. Informed consent forms were produced and signed by home owners and catchers at each site. The protocol for identifying the host blood was approved by the Scientific Ethics Committee of Marseille (C2EA-14) and by the French authorities.

### Mosquito rearing

*An*. *gambiae* Giles were reared at the insectarium in our laboratory using standard methods [12]. The larvae were kept in water until the nymph stage and fed fish food (TetraMinBaby, Tetra Gmbh, Herrenteich, Germany). Pupae were collected using a dropper and placed into plastic receptacles, and then transferred to cages (Bug Dorm 1; Bioquip Products). The emerging adults were fed on 10% glucose until the day of the experiment. The mosquitoes were kept in standard insectary conditions at 26±2°C, 80% relative humidity and with a 12:12 hour light/dark cycle. Three days after emerging, the female adults were used in the experiment.

### Experiment

#### Bloody Whatman filter paper (BWFPs) process

In the first phase of this study, twenty (n = 20) three-day-old mosquitoes were artificially engorged on human or sheep blood. *An*. *gambiae* Giles females were fed through a Parafilm-membrane (hemotek membrane feeding systems, Discovery Workshops, UK) as described [12]. The engorged females were harvested 12 hours after feeding and were anaesthetized at −20°C for 10 minutes. The abdomens of 10 mosquitoes per host blood source (human or sheep), were first individually crushed on Whatman filter papers (Whatman International Ltd. Maidstone England, approved by BSI) and maintained at ambient temperature for a maximum of four hours (drying time). In order to crush the mosquito abdomen on Whatman filter paper, the engorged females were individually deposited on a card. Sterile forceps were used to apply pressure to release the blood meals from the mosquito abdomens. After discharge of the blood meals onto the filter paper, the blood deposits were triturated on the card with a sterile piston. The Whatman paper containing the blood was then individually air dried for four hours before being submitted for MALDI-TOF analysis and different storage conditions. They were then used for MALDI-TOF MS analysis. In the second phase, ten mosquitoes were fed on one of four vertebrate host bloods: rabbit, dog, chicken and rat. The abdomens of ten mosquitoes per host blood were crushed on WFPs for MALDI-TOF analysis.

#### Storage of bloody Whatman filter papers

Three storage methods were tested: room temperature, +4°C, and −20°C (frozen group). The mosquitoes used for this part of our study were engorged on cow or goat blood. The engorged females were harvested 12 hours after feeding. Each preservation method was applied to 50 *An*. *gambiae* Giles bloody WFPs. For each storage method, five specimens per host blood WFP were individually submitted for MALDI-TOF MS analysis after 7, 14, 30, 60 and 90 days.

### Mosquito collection in the field

The field part of the study was carried out in the Comoros archipelago situated at the northern entrance of the Mozambique Channel, off the eastern coast of Africa, between northeastern Mozambique and northwestern Madagascar. The mosquitoes were captured on three islands (Ngazidja, Moheli and Anjouan) of the Comoros archipelago (11° 20' to 13° 4' S and 42° to 45° E) [19], between March and August 2015. The mosquitoes were collected by human landing catch. All specimens were captured either inside or outside domestic buildings. Mosquitoes were morphologically identified using the adult species determination key [20,21]. The engorged female abdomens were crushed on WFPs and preserved at -20°C, before being used for MALDI-TOF MS analysis in Marseille, France, in April 2016.

### Whatman filter paper sample preparation for MALDI-TOF analysis

The pieces of bloody WFP containing the crushed abdomen of the engorged mosquito (about 1 mm^2^) were individually cut using a sterile scalpel and transferred to a 1.5 mL tube. Two protein elution methods were used for test preparation.

In the first elution method, 20 μL of formic acid (FA) (70% v/v) and 20 μL of acetonitrile (ACN) (50% v/v; Fluka, Buchs, Switzerland) was added and incubated for 10 minutes at room temperature (RT). The benefit of this protocol is the speed of sample preparation for MALDI-TOF analysis (protocol 1). In the second method, pieces of bloody WFPs were incubated for 10 minutes in 50 μL of high performance liquid chromatography (HPLC) grade water. Subsequently, 10 μL of the homogenized substance from the crushed bloody WFPs of the incubated mosquito abdomens was added to 40 μL of organic buffers (FA/ACN) (protocol 2).

After a fast spin (10,000 rpm for 20 seconds), 1 μL of supernatant of each elution method sample was loaded onto the MALDI-TOF target plate in quadruplicate (Bruker Daltonics, Wissembourg, France) and covered with 1 μL of CHCA matrix solution composed of saturated α-cyano-4-hydroxycynnamic acid (Sigma, Lyon, France), 50% acetonitrile (v/v), 2.5% trifluoroacetic acid (v/v) (Aldrich, Dorset, UK) and HPLC-grade water. After drying for several minutes at RT, the MALDI-TOF target plate was introduced into the Microflex LT MALDI-TOF Mass Spectrometer (Bruker Daltonics, Germany) for analysis. As a control for the mass spectra steel loading, matrix quality and MALDI-TOF apparatus performance, a matrix solution was loaded in duplicate onto each MALDI-TOF plate with and without a bacterial test standard (Bruker Bacterial Test Standard, ref: #8255343). In addition, 1 μL of blood and dissected mosquito abdomens from engorged specimens of that host were processed as previously described [12] and loaded in quadruplicate onto the MALDI plate. The MALDI-TOF MS set up parameters were similar to those previously described [12].

### Spectra analysis

All MS spectra from bloody WFPs with crushed abdomens from mosquitoes fed on human, sheep, rat, rabbit, dog and chicken blood collected at 12 hours after feeding were viewed and analyzed using Flex analysis v.3.3 software. To determine reproducibility and specificity, the bloody WFP MS spectra from the preliminary and secondary phases of this study were aligned with homologous MS spectra of intact abdomens available in the database. The alignment was performed by Flex analysis and using ClinProTools 2.2 (Bruker Daltonics) software. We performed a comparison of MS spectra obtained from our previous work using intact engorged mosquito abdomens with those obtained in this study from abdomens crushed on WFPs to determine the MS spectrum stability of bloody WFPs. The software (Bruker Daltonics) was used to compare the average spectra obtained from the four spectra of each sample.

### Blind tests

MALDI-TOF MS spectra obtained from engorged mosquito abdomens crushed on WFPs were evaluated against our home-made arthropod database. This database has been upgraded since our proof-of-concept report [12]. The database includes the spectra obtained from (i) the intact abdomens of mosquitoes engorged on 17 host bloods (*Homo sapiens*, *Equus caballus*, *Ovis aries*, rabbit, Balb/C mouse, *Rattus norvegicus*, *Canis familiaris*, *Bos taurus*, *Capra hircus*, *Gallus gallus*, *Equus asinus*, *Tapirus indicus*, *Tapirus terrestris*, *Carollia perspicillata*, *Thraupis episcopus*, *Erythrocebus patas* and *Callithrix pygmaea*), (ii) bloody WFPs obtained from mosquito abdomens crushed after feeding on human or sheep blood, and (iii) various sections from 30 mosquito species (*Anopheles gambiae*, *An*. *coluzzi*, *An*. *funestus*, *An*. *ziemanni*, *An*. *arabiensis*, *An*. *wellcomei*, *An*. *rufipes*, *An*. *pharoensis*, *An*. *coustani*, *An*. *claviger*, *An*. *hyrcanus*, *An*. *maculipennis*, *Culex quinquefasciatus*, *Cx*. *pipiens*, *Cx*. *modestus*, *Cx*. *insignis*, *Cx*. *neavei*, *Aedes albopictus*, *Ae*. *excrucians*, *Ae*. *vexans*, *Ae*. *rusticus*, *Ae*. *dufouri*, *Ae*. *cinereus*, *Ae*. *fowleri*, *Ae*. *aegypti*, *Ae*. *caspius*, *Mansonia uniformis*, *Orthopodomyia reunionensis*, *Coquillettidia richiardii* and *Lutzia tigripes*), sandflies (six species: *Phlebotomus papatasi*, *P*. *(Larrousius) longicuspis*, *P*. *(Larrousius) perfiliewi*, *P*. *(Larrousius) perniciosus*, *P*. *(Paraphlebotomus) sergenti*, *Sergentomyia minuta*), triatomines (six species: *Triatoma infestans*, *Rhodnius prolixus*, *Rh*. *pictipes*, *Rh*. *robustus*, *Eratyrus mucronatus*, *Panstrongylus geniculatus*), ticks (17 species: *Amblyomma cohaerens*, *Am*. *gemma*, *Am*. *variegatum*, *Haemaphysalis leachi*, *Hyalomma marginatum rufipes*. *H*. *truncatum*, *H*. *detritum*, *Rhipicephalus decoloratus*, *Rh*. *bergeoni*, *Rh e*. *evertsi*, *Rh*. *praetextatus*, *Rh*. *pulchellus*, *Rh*. *sanguineus*, *Rh*. *microplus*, *Rh*. *annulatus*, *Rh*. *turanicus*, *Rh*. *bursa*), mites (three species: *Leptotrombidium chiangraiensis*, *L*. *imphalum*, *L*. *deliense*), bedbugs (one species: *Cimex lectularius*), louse (7 species: *Pediculus humanus*, *Damalinia bovis*, *D*. *caprae*, *D*. *ovis*, *Haematopinus eurysternus*, *Linognatus vituli*, *L*. *africanus*) and fleas (five species: *Ctenocephalides felis*, *Ct*. *canis*, *Archaeopsylla erinacei*, *Xenopsylla cheopis* and *Stenoponia tripectinata*). The level of significance was determined using the log score values (LSVs) given by the MALDI-Biotyper software v.3.3, corresponding to matching query and reference mass spectra signal intensities. LSVs ranged from zero to three. A sample was considered to be correctly and significantly identified at the species level when the queried spectrum had a log score value (LSV) ≥ 1.8 [12,22,23].

### Identification of field mosquito blood meals

All bloody WFPs obtained in the field from engorged mosquitoes collected on Comoros were tested by MALDI-TOF MS as described above. To confirm the identification of the mosquito blood meal by MALDI-TOF, molecular identification was attempted on bloody WFPs which were randomly selected (n = 30) from the Comoros samples. DNA extractions from Whatman papers with individual mosquito abdomen samples were performed with the EZ1 DNA Tissue kit (Qiagen, Hilden, Germany) as per the manufacturer’s instructions. DNA extracted from unfed mosquitoes was used as a negative blood meal control. A set of the primers specifically amplifying a fragment of 648 bp for the vertebrate Cytochrome c oxidase I gene (vCOI) was used and the PCR reaction was conducted as described [24]. vCOI positive PCR products were purified using the NucleoFast 96 PCR plate (Machery-Nagel EURL, France), and sequenced using the same primers with the BigDye version 1–1 Cycle Ready Reaction Sequencing mix (Applied Biosystems, Foster City, CA, USA) and an ABI 3100 automated sequencer (Applied Biosystems) to control the amplified products. The sequences were assembled and analyzed using the ChromasPro software (version 1.34) (Technelysium Pty. Ltd., Tewantin, Australia) and BLAST website (Basic Local Alignment Search Tool; http://blast.ncbi.nlm.nih.gov).

## Results

### Bloody WFP MALDI-TOF MS analysis set up

**Fig 1:**
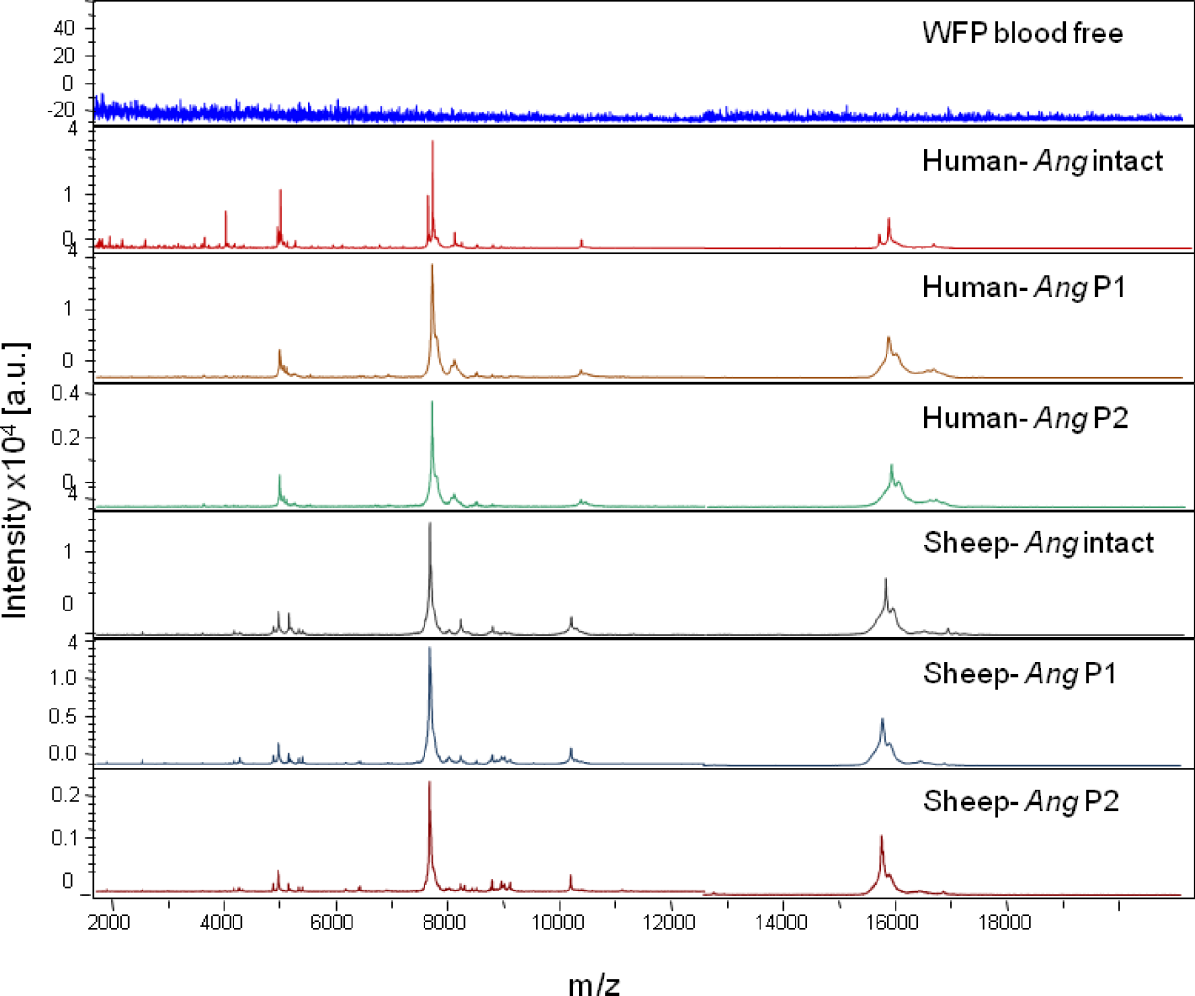
MALDI-TOF MS spectra from *An*. *gambiae* Giles (*Ang*) abdomen protein extracts engorged on vertebrate host bloods and then crushed on Whatman filter papers (WFPs). The MS spectra were generated according to two protocols (P1, P2). Intact *Ang* match the MS profiles from *Anopheles gambiae* Giles abdomens crushed on WFPs. The blood-free WFP corresponds to the MS profiles of WFPs where no mosquito blood meal was released. The vertebrate host bloods used for *Anopheles gambiae* Giles bloody Whatman filter papers (bloody WFPs) were human and sheep. All mosquitoes were collected 12 hours after feeding. a.u. arbitrary units; m/z mass-to-charge ratio.

The MS spectra from WFPs with and without blood from crushed abdomens were visually analyzed using Flex analysis software (Fig 1). The spectrum of blood-free WFPs had no peaks. The organic buffer and WFPs used as negative controls did not generate any peaks. The comparison of the MS spectra obtained from bloody (human and sheep) WFPs and those obtained from *Anopheles* engorged abdomens (human and sheep) showed many similar peaks for the same host blood source (Fig 1). The MS spectra of bloody WFPs from specimens fed on human and sheep blood were also compared using the elution method (protocol 1 and protocol 2). The representative spectra of the two bloody WFPs processed with protein elution methods revealed high peak intensities (Fig 1). All MALDI-TOF MS profiles from bloody mosquito WFPs using Flex analysis software clearly showed a reproducible spectrum by blood meal origin (Fig 1). When twenty bloody WFPs were tested against our database, the blind test yielded a 100% correct identification of blood origin. The blind test revealed log score values (LSVs) greater than 1.8 (Table 1). Therefore, the rapid protocol using only organic buffers (protocol 2) was used for all subsequent processing.

**Table 1:** Blind tests of the *An*. *gambiae* Giles used to set up the protocol optimal for blood meals identification by MALDI TOF from BWFPs.

Mosquito species	Blood feeding source	Number of specimens spotted used blind tests against database (number per elution mode)	High LSVs obtained from blind tests against database ^(a)^	Vertebrate species identification of blood origin
*An*. *gambiae* Giles	Human	10 (5/5)	[1.985–2.930] (10)	Human
*An*. *gambiae* Giles	Sheep	10 (5/5)	[1.845–2.259] (10)	Sheep
Total		20		

All mosquitoes were collected 12 hours post-feeding to crushing in the whatman filter

^(a)^Into brackets were indicated the number of MS spectrum tested against the database with a LSVs upper 1.8 considered significative

### Assessment of MALDI-TOF MS using bloody WFPs from *Anopheles gambiae* Giles fed on combination host blood meals

**Fig 2:**
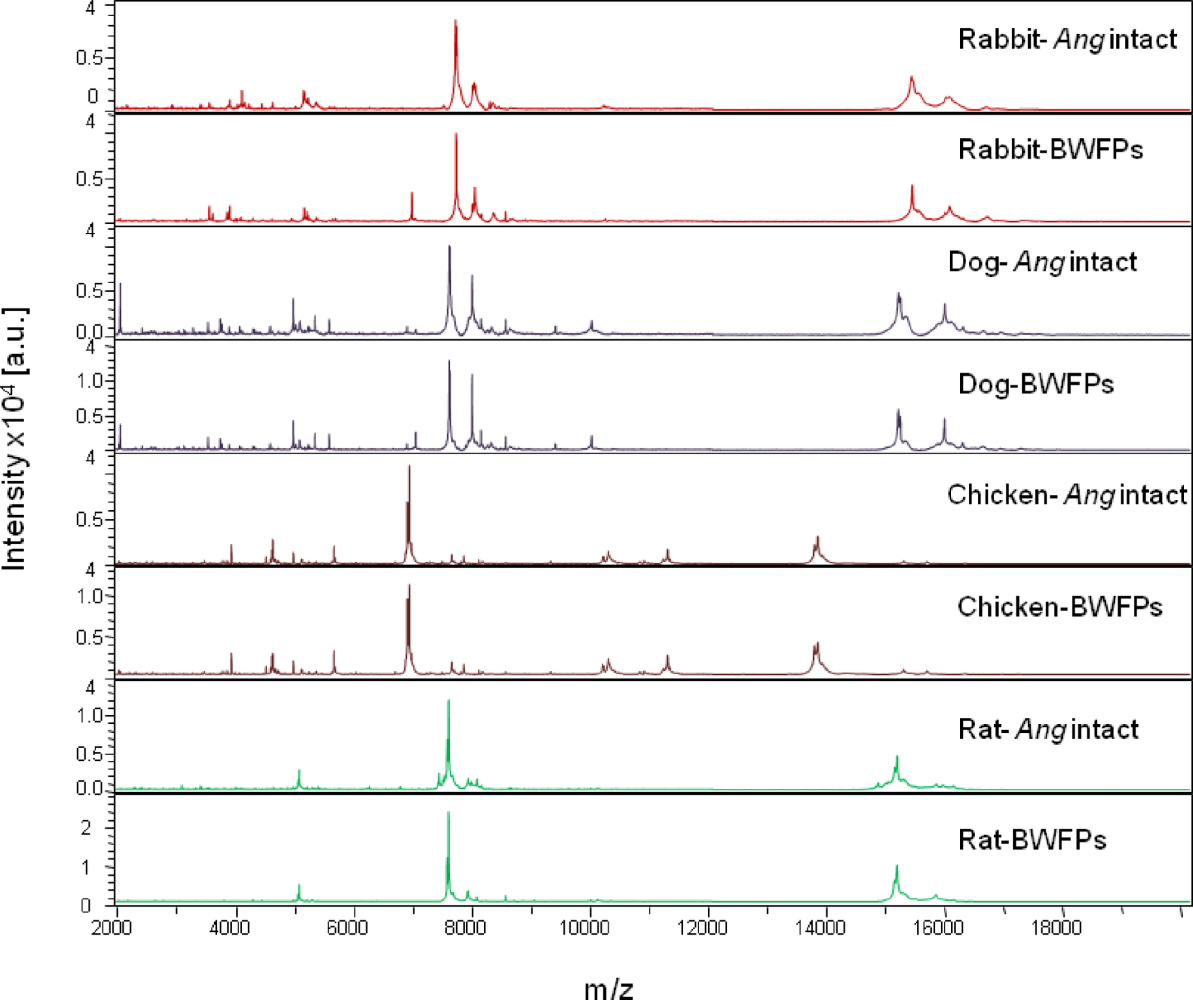
Comparison of MALDI-TOF MS spectra from *An*. *gambiae* Giles abdomen protein extracts engorged on vertebrate host bloods and then crushed on Whatman filters. The MS spectrum alignment was performed by Flex analysis 3.3 software. Intact *Ang* match the MS profiles from *Anopheles gambiae* Giles abdomens crushed on WFPs. The vertebrate host bloods used for mosquito blood meals were rabbit, dog, chicken and rat. All mosquitoes were collected 12 hours after feeding. a.u. arbitrary units; m/z mass-to-charge ratio.

After the above set-up was complete, 40 bloody WFPs from engorged *Anopheles gambiae* Giles fed on rabbit, dog, rat and chicken blood were submitted for MALDI-TOF MS analysis. As the bloody WFP MS spectra were previously reported to be stable, comparison of the 40 bloody WFP MS spectra by Flex analysis software indicated a high reproducibility and specificity by blood meal origin (Fig 2). The bloody WFPs from crushed engorged mosquito abdomens fed on the same host were perfectly superimposable. When we tested the MALDI-TOF MS spectra obtained from the 40 bloody WFP abdomens against our MALDI-TOF MS database, the blind test revealed a 100% correct identification of the host blood source in all mosquitoes tested. The log score values (LSVs) ranged from 1.918 to 2.841 (Table 2). A comparison of the MS spectra of crushed abdomen bloody WFPs with those of intact engorged mosquito abdomens loaded in our database indicated great similarity for mosquitoes which were fed on the blood of the same animal. The alignment reveals intra-species reproducibility and inter-species specificity (Fig 2).

**Table 2:** *An*. *gambiae* Giles abdomen crushing in paper filter Whatman to performed the blind tests according blood meal source.

Mosquito species	Blood feeding source	Number of specimens spotted used blind tests against database	High LSVs obtained from blind tests against database ^(a)^	Vertebrate species identification of blood origin
*An*. *gambiae* Giles	Rabbit	10	[1.918–2.188] (10)	Rabbit
*An*. *gambiae* Giles	Dog	10	[2.382–2.728] (10)	Dog
*An*. *gambiae* Giles	Rat	10	[1.981–2.481] (10)	Rat
*An*. *gambiae* Giles	Chicken	10	[2.662–2.841] (10)	Chicken
Total		40		

All mosquitoes were collected 12 hours post-feeding to crushing in the whatman filter

^(a)^Into brackets were indicated the number of MS spectrum tested against the database with a LSVs upper 1.8 considered significative

### Whatman filter storage method for mosquito blood meal identification

**Fig 3:**
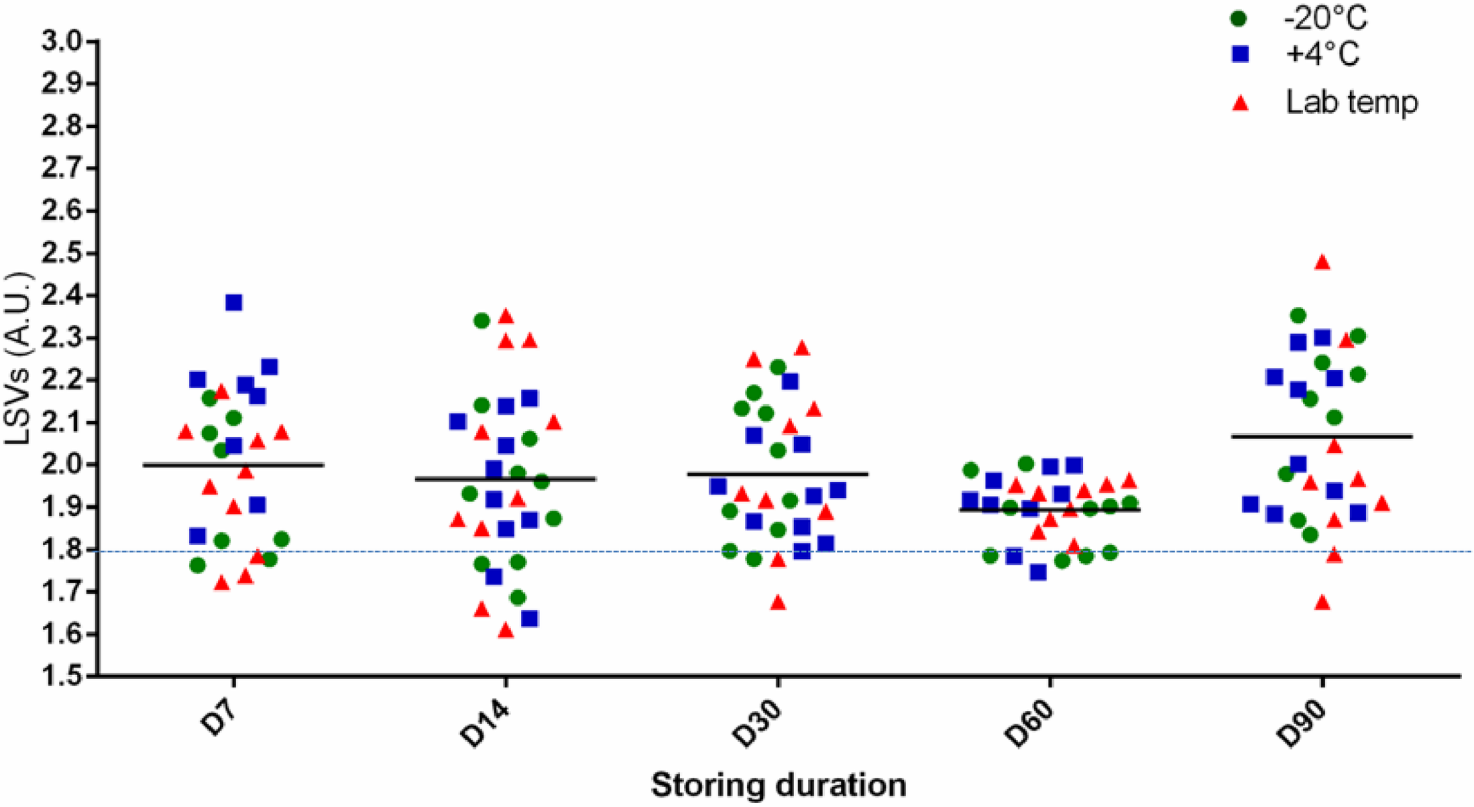
Whatman filter storage method for mosquito blood meal identification. Comparison of LSVs obtained following a reference TP database query with MS spectra of *An*. *gambiae Giles* fed on cow and goat blood. All specimens were collected 12 hours after feeding and stored up to 90 days (D). The mosquito abdominal proteins crushed on WFPs were stored under three different conditions: -20°C, + 4°C and room temperature. The dashed line represents the threshold value for relevant identification (LSVs >1.8). LSV, log score value.

When five bloody WFPs per storage method (-20°C, + 4°C and room temperature) were processed with MALDI TOF MS analysis at a given time point (7, 14, 30, 60 and 90 days), the blind test performed against the database reliably and correctly identified 100% of the bloody WFP (n = 150) mosquitoes tested up to three months. The log score values (LSVs) for these MS bloody WFPs ranged from 1.612 to 2.481 (with 134/150 > 1.8) (Fig 3). Visually, the MS spectra from the stored BWFPs remained stable up to 90 days post-collection (Fig 3). To this end, MS spectra comparison by Flex analysis software between the three storage methods indicated similar profiles quality following these storing modes.

### Identification of field mosquito blood meals

A total of 216 mosquitoes collected in Comoros were morphologically identified as: 93 *An*. *gambiae s*.*l*. and 123 *An*. *funestus s*.*l*. A total of 77 females were found to be engorged, including 54 *An*. *gambiae s*.*l*. and 23 *An*. *funestus s*.*l*. The engorged female abdomens were crushed on WFPs.

Twelve months later, in Marseille, the bloody WFPs were submitted for MALDI-TOF MS analysis to identify the blood meals. The 77 MS spectra obtained from the bloody WFPs were queried against our MALDI-TOF MS database. The blind test reveals that 70/77 MS spectra from Comoros submitted for MALDI TOF analysis matched human blood with high LSV scores (1.818–2.690) (Table 3). The seven remaining MS spectra from Comoros were too low and or of too poor quality to be identified by MALDI-TOF MS. A total of 27 COI sequences were obtained from Comoros bloody WFP samples. The amplified fragment sequences were shown to share between 99.11% and 99.85% similarity with several sequences of human COI (Genbank Accession: KX457614.1, MF278749.1).

**Table 3:** Blind tests of Comoros mosquitoes to identified their blood meals sources by MALDI-TOF MS from whatman paper against TPDB.

Sites	Samples tested	location	Morphological identification of mosquito	Vertebrate species of blood origin	Number of correct matching	High LSVs from blind tests database	Date of collect
Anjouan(Chirove)	16	indoor	*Anopheles gambiae*	Human	16	[2.005–2.553]	01/03/2015
Anjouan(Chirove)	1	indoor	*Anopheles funestus*	Low quality	/	/	01/03/2015
Moheli(Hamavouna)	1	indoor	*Anopheles gambiae*	Human	1	2.124	01/03/2015
Anjouan(Kowe)	1	indoor	*Anopheles gambiae*	Human	1	2.252	01/03/2015
Ngazidja(Singani)	1	indoor	*Anopheles gambiae*	Human	1	2.435	01/03/2015
Ngazidja(Wela)	1	indoor	*Anopheles gambiae*	Low quality	/	/	01/03/2015
Ngazidja(Wela)	3	indoor	*Anopheles gambiae*	Human	3	[1.869–2.239]	01/03/2015
Ngazidja(Chamle)	1	outdoor	*Anopheles gambiae*	Human	1	2.141	01/03/2015
Anjouan(Chirove)	9	outdoor	*Anopheles gambiae*	Human	9	[1.915–2.672]	01/03/2015
Moheli(Hamavouna)	2	outdoor	*Anopheles gambiae*	Human	2	[1.818–2.200]	01/03/2015
Ngazidja(Singani)	1	outdoor	*Anopheles gambiae*	Low quality	/	/	01/03/2015
Moheli(Fomboni)	1	*	*Anopheles gambiae*	Human	1	2.150	01/03/2015
Moheli(Hamavouna)	1	*	*Anopheles gambiae*	Human	1	2.095	01/03/2015
Ngazidja(Wela)	1	indoor	*Anopheles gambiae*	Human	1	2.218	01/03/2015
Anjouan(Kowe)	1	outdoor	*Anopheles gambiae*	Human	1	2.319	01/03/2015
Ngazidja(Wela)	3	outdoor	*Anopheles gambiae*	Human	3	[2.152–2.315]	01/03/2015
Anjouan(Chirove)	1	*	*Anopheles gambiae*	Human	1	2.124	01/03/2015
Moheli(Fomboni)	1	*	*Anopheles gambiae*	Human	1	2.333	01/03/2015
Moheli(Hamavouna)	1	*	*Anopheles gambiae*	Low quality	/	/	01/03/2015
Moheli(Hamavouna)	1	*	*Anopheles gambiae*	Low quality	/	/	01/03/2015
Moheli(Hamavouna)	1	*	*Anopheles gambiae*	Human	1	2.249	01/03/2015
Moheli(Wela)	1	*	*Anopheles gambiae*	Human	1	2.285	01/03/2015
Anjouan(Chirove)	15	indoor	*Anopheles funestus*	Human	15	[1.910–2.690]	01/03/2015
Anjouan (Chirove)	1	indoor	*Anopheles funestus*	Low quality	/	/	01/08/2015
Anjouan (Chirove)	5	outdoor	*Anopheles funestus*	Human	5	[1.969–2.639]	01/08/2015
Anjouan (Chirove)	1	outdoor	*Anopheles funestus*	Low quality	/	/	01/08/2015
Anjouan (Chirove)	1	outdoor	*Anopheles funestus*	Human	1	2.614	01/08/2015
**Total**	77				70		

## Discussion

The major advantages of MALDI-TOF MS technology include the rapidity, accuracy and low cost of analyses (under US$1 per sample) [25,26]. This may revolutionize the entomological domain. Limitations of this method of specimen identification include the cost of the device and database comprehensiveness. However, when the MALDI TOF device is bought for clinical microbiology purposes, it can also be used for medical entomology at no additional cost. This is what has happened in Dakar, Senegal, where the Hôpital Principal de Dakar laboratory is now equipped with MALDI-TOF devices for clinical microbiology purposes [27], and entomological applications have already been developed, with MALDI-TOF identification of Culicoides [28].

Having previously reported the proof-of-concept for MALDI-TOF’s ability to identify the blood meals of mosquitoes by analyzing their engorged abdomens, here we report another promising application in the field of entomology. WFPs are the most common way of collecting and preserving mosquito blood meals in the field. Since a database of several animal blood spectra obtained from engorged mosquitoes has already been created, in this study we used the Whatman filter as a as standard technique for field sample storing and transport during entomological investigations.

Our experiment was performed using bloody WFPs from *An*. *gambiae* Giles blood meals submitted for MALDI-TOF MS analysis. Interestingly, only a small portion of bloody WFP (about 1mm^2^) was required to identify the blood meal source. The remaining BWFP can be used for further analysis, such as the molecular detection of plasmodium [29]. The rapidity of the analysis time makes it feasible to identify the origins of the mosquito blood meal using MALDI TOF MS. Only ten minutes per sample was necessary between the crushing process and identification of the blood meals.

After our preliminary tests, we chose to process bloody WFPs treated with organic buffers (protocol 2), as it was a simple and effective means of generating MALDI-TOF MS spectra of good quality. It also delivered intra-species reproducibility and inter-species specificity of the origins of mosquito blood meals. One advantage of using water instead of a cocktail of formic acid and acetonitrile is that remains of the host blood can be used for other purposes and techniques, including molecular tools. Our experimental protocol performed on a wide range of animal bloods (rabbit, dog, rat and chicken) confirmed that analyzing WFPs with crushed *An*. *gambiae* Giles engorged abdomens yielded spectra with intra-species reproducibility and inter-species specificity. The blind test positively supported the ability of MALDI-TOF MS to identify blood meal sources using WFPs, with high LSVs up to 2.930 (Table 1), when 1.8 had previously been estimated as a reliable threshold for arthropod identification [22,23] and mosquito blood meal identification[12].

During transport from the field to the laboratory, sample storage issues are critical, even when sampling has been adapted to field conditions, such as using WFPs to collect blood or the blood contained in engorged mosquitoes. In our study, the preservation method (- 20°C, + 4°C and room temperature) did not affect the identification of mosquito blood meals by MALDI-TOF MS. The stability of the MS profiles from all preservation methods had LSVs over 1.8 for up to three months, which suggests that WFPs are robust and sufficient for preserving field collection samples. This hypothesis was tested on bloody WFPs obtained from mosquitoes collected in Comoros. In Comoros, the bloody WFP samples and all collected mosquitoes were preserved by freezing. The bloody WFPs were transported from Comoros to Marseille at ambient temperature before undergoing MALDI-TOF MS analysis. The time between collecting bloody WFPs from Comoros and performing MALDI TOF MS analysis was about one year.

In Comoros, WFPs are commonly used to store blood for malaria studies [30]. Malaria is an endemic disease in Comoros and has long been considered a major public health issue with prevalence over 35% in 2009 in the Union of Comoros. Malaria vectors are *An*. *gambiae s*.*l*. and *An*. *funestus s*.*l*. [20]. *An*. *gambiae* is present on the third island while *An*. *funestus s*.*l*. is only present in Anjouan and Moheli [31]. Different strategies have been used by the Malaria Control Program to control both parasites and vectors. The parasitological study carried out during the Malaria Indicator Survey (MIS) in June 2014 showed a significant reduction in prevalence to under 1% in the three islands of the Union of the Comoros, with 1.4% in Grande Comore, 0.5% in Anjouan and 0% in Moheli [32]. However, no entomological studies have been conducted to assess the impact of these operations on malarial transmission in the country. We took the opportunity presented by an entomological survey conducted by the PNLP (Programme National de Lutte contre le Paludisme) of Comoros to test MALDI TOF MS’s ability to identify the blood meals of mosquitoes using WFPs. Results were very convincing, with 90% of the Comoros engorged mosquito abdomens crushed on WFPs being undoubtedly identified by MALDI TOF MS as human blood sources. The low quality of the seven MS spectra generated by MALDI-TOF MS analysis can be attributed to the MS profile protein alteration [33], as we found in our previous study that MALDI-TOF MS can identify blood meals up to 24 hours after feeding. However, some studies indicate that MS spectra modifications are due to storage methods, such as storing specimens in alcohol [12], or due to extraction models [34]. Apparently, alcohol conservation rapidly modifies the MS spectra of mosquito abdomens fed on different mammals after only two weeks of storage [12]. The results of the blood meal identification of engorged females collected by human land catch in Comoros might be considered surprising. Indeed, the female mosquito is supposed to not have the time to feed on the exposed legs of collectors. However, these mosquitoes may have started to feed on other human hosts before landing on the collectors. Also, malaria has been reported in collectors exposed to human land catch, although the incidence is much lower than in the local population [35]. We used COI sequence analysis, as vertebrate cytochrome c oxidase I gene has a low variability within species [36,37] and might serve as a DNA barcode for the identification of animal species [38].

## Conclusions

Our results show the correct identification of anopheles vector blood meal sources (field catch and laboratory reared) by MALDI-TOF MS using WFPs. The future use of MALDI TOF in mosquito studies may include to identify the mosquito from legs [13] and the blood meal from the abdomen, and also detect *Plasmodium* from the cephalothoraxes [39]. Our database could subsequently be shared and open access could be granted for routine arthropod identification either for entomological diagnosis or arthropod monitoring. Other future developments in the field of blood meals include identifying the blood meal mixture, parity status and mosquito age.
